# Testing of the 4SM Method in the Gulf of California Suggests Field Data Are not Needed to Derive Satellite Bathymetry

**DOI:** 10.3390/s17102248

**Published:** 2017-09-30

**Authors:** Fabio Favoretto, Yann Morel, Andrew Waddington, Jorge Lopez-Calderon, Marco Cadena-Roa, Anidia Blanco-Jarvio

**Affiliations:** 1 Posgrado en Ciencias Marinas y Costeras (CIMACO), Universidad Autónoma de Baja California Sur, 23060 La Paz, Baja California Sur, Mexico; favoretto.fabio@gmail.com; 2 Retired, BP 2862 98703 Puanaauia, French Polynesia; rspfsm@watercolumncorrection.com; 3 AW Hydrographic, 35 Burge Meadow, Cotford St. Luke, Taunton Somerset TA4 1QN, UK; A.Waddington@awhyd.co.uk; 4 Facultad de Ciencias Marinas, Universidad Autónoma de Baja California, , Carretera Tijuana-Ensenada 2917, Col. Playitas, C.P. 22860 Ensenada B.C., Mexico; jorge.lopez67@uabc.edu.mx; 5 Programa de Bioingeniería y Ciencias Ambientales, Departamento Académico de Ingeniería en Pesquerías, Universidad Autónoma de Baja California Sur, Unidad Pichilingue, 23060 La Paz, Baja California Sur, Mexico; marco@uabcs.mx

**Keywords:** 4SM, satellite derived bathymetry, water depth, water column correction, remote sensing, Landsat, San Lorenzo Channel

## Abstract

Satellite-derived bathymetry methods over coastal areas were developed to deliver basic and useful bathymetry information. However, the process is not straightforward, the main limitation being the need for field data. The Self-calibrated Spectral Supervised Shallow-water Modeler (4SM) method was tested to obtain coastal bathymetry without the use of any field data. Using Landsat-8 multispectral images from 2013 to 2016, a bathymetric time series was produced. Groundtruthed depths and an alternative method, Stumpf’s Band Ratio Algorithm, were used to verify the results. Retrieved (4SM) vs groundtruthed depths scored an average r^2^ (0.90), and a low error (RMSE = 1.47 m). 4SM also showed, over the whole time series, the same average accuracy of the control method (40%). Advantages, limitations and operability under complex atmosphere and water column conditions, and high and low-albedo bottom processing capabilities of 4SM are discussed. In conclusion, the findings suggest that 4SM is as accurate as the commonly used Stumpf’s method, the only difference being the independence of 4SM from previous field data, and the potential to deliver bottom spectral characteristics for further modeling. 4SM thus represents a significant advance in coastal remote sensing potential to obtain bathymetry and optical properties of the marine bottom.

## 1. Introduction

Accurate bathymetry in the coastal area is one of the most basic yet fundamental sources of information for many purposes like navigation, military, resource exploitation, fisheries and tourism. Water depth is also a major factor in community zonation and is a fundamental variable to map and model benthic habitats, for research and conservation purposes. In marine sciences, acoustic, radar and optical systems have been used to assess bathymetry [1]. However, they require substantial time and effort to be deployed, operated and interpreted. Such an effort can be challenging, especially in developing countries. Furthermore, vast regions are too costly or difficult to reach for these conventional methods: this creates gaps in the knowledge of underwater habitats. Recently, satellite technologies have developed rapidly and the NASA-USGS Landsat Data Continuity Mission [2] offers a unique opportunity to get open-source multispectral images (https://earthexplorer.usgs.gov). Landsat-8 is the newest multispectral satellite developed from the Landsat series which have been used to identify benthic objects in shallow waters [3,4,5,6,7,8,9]. Landsat-8 features a higher spectral resolution and quality than its predecessors. The availability of images makes Landsat-8 the main open source alternative for coastal monitoring. However, the challenge is to obtain the underwater optical properties which are required to model bathymetry and bottom reflectance.

Among the several factors that determine the radiance spectrum recorded by the sensor, four main issues require suitable “correction” in order to retrieve acceptable results: (1) atmospheric correction to remove the diffusion of photons through the atmosphere; (2) sun and sky glint correction to remove the specular reflection of photons at the air-water interface; (3) water column correction to remove the exponential decay caused by the diffusion and attenuation of photons through the shallow water column; and finally (4) an assumption on the bottom reflectance to account for the reflection of photons on the bottom [10]. The water column correction algorithms published to date have been extensively reviewed [10,11]. They differ in the way of estimating the several contributions to the water leaving spectral signature, and all of them require field data for calibration purposes. They are based on the knowledge of the physics involved (analytical), or on field data (empirical), or both (semi-analytical). The most popular among empirical methods are band combination algorithms, which are simple and easy to use; they assume that water leaving reflectance in multispectral bands is an exponential function of bottom substrate reflectance, bottom depth, and diffuse attenuation coefficient. The water leaving signal over a shallow bottom is a function of the bottom depth and reflectance, and also of the optical properties of the water columnlike reflectance over deep water (Lw) and the diffuse attenuation coefficient (Kd). These are essential parameters for water column correction methods [10], and have also been used to classify water types [12], estimate chlorophyll concentrations [13], predict light intensity at depth [14], estimate the rate of solar heating in surface waters [15]. Since the depth at each pixel is constant for each multispectral band of the satellite image, the relationship between radiance in two distinct bands can be used to estimate Kd. Estimating Kd from field data is difficult; it can be approximated using various band ratio methods [16,17,18,19]. The first and most popular [16], assumes that differences of a group of pixels on the same substrate are due to depth variation and not another factor. This can be true in small areas, but if the area is large, or the bottom type is heterogeneous, the only solution is a calibration with ground-truthed Kd measurements [20]. Moreover, Lyzenga’s algorithm does not retrieve substrate reflectance, while the algorithm removes most of the depth effects, it generates a depth-invariant bottom index which is not related to the physics behind [10]. Lyzenga et. al. [21] further enhanced the methodology using multiple linear regressions which, however, still requir knowledge of water’s optical properties at the time of image acquisition, which is not always available nor easy to obtain. Stumpf et al. [22] presented a Log Ratio method to account for variable bottom types, which has been used since then by NOAA for coastal mapping and is available in an image processing software (ENVI, and SPEAR toolbox). This method still requires existing depth measurements for calibration and does not produce a water column corrected image. The method developed by Sagawa et al. [19] for low transparency waters requires depth and attenuation coefficient observations measured in the field. It may be valid only within small areas and authors emphasize the need for an accurate sea-truthed map to obtain results. Therefore, it does not produce an estimation of retrieved bottom reflectance.

In contrast with all other methods in use to-date, the Self-calibrated Spectral Supervised Shallow water Modeler (4SM) [23], does not rely on preliminary sets of field data for the calibration of the model parameters. 4SM was first presented by Morel and Lindell [24] and is explained in detail by Morel and Favoretto [23] and at http://www.watercolumncorrection.com/. We tested the 4SM approach to process a time series of nine Landsat-8 images without the use of any field data for the calibration of the simplified radiative transfer equation (RTE). Furthermore, we compare the results with those obtained using the Log Ratio method and with a depth sounding dataset. The potential of 4SM to retrieve depths from multispectral images without the use of field data is a significant advance in the exploration and monitoring potentials in coastal areas.

## 2. Materials and Methods 

### 2.1. Study Area

**Figure 1:**
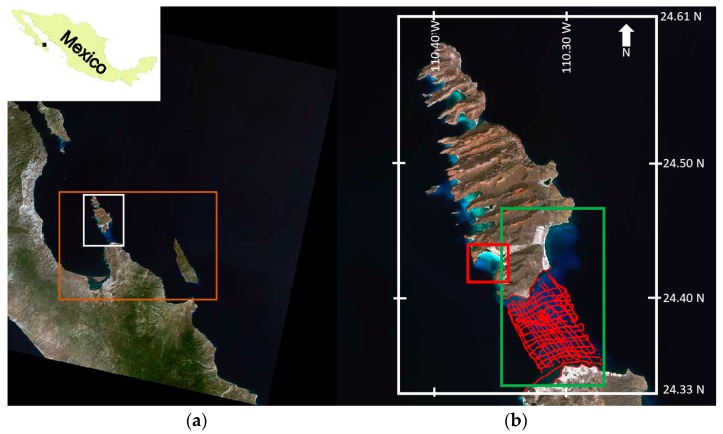
Landsat scene: LC80340432013292LGN00. (**a**) whole image with study area (orange rectangle), and Espiritu Santo island and the SLC (white rectangle); (**b**) red square is the area used for parameter calibration, and green rectangle is the SLC.

The San Lorenzo channel (SLC) was chosen as study area for its morphology and for the variety of its shallow habitats [25,26]. The SLC is a shallow area that connects the Bay of La Paz to the Gulf of California (Figure 1). The communication between the Bay and the Gulf of California happens also through the “Boca Norte” (Northern mouth): this large opening reaches a depth of 350 m. The region has two contrasting seasons: a dry season (October-June) and a humid season (July–September); the latter is referred to as “Mexican monsoon” [27] and is associated with occasional significant precipitation, often through intense events like hurricanes. Hydrographic conditions in the bay, particularly in deeper areas, are strongly influenced by the seasonal variability of main thermohaline circulation of the southern Gulf of California [28]. Salinity ranges between 34‰ and 36‰ depending on the time of the year [29]. In particular, the deeper layers, are more influenced by the Gulf of California, while shallower ones are dominated by local processes like insolation, evaporation, precipitation and mixing [30]. Because of the high solar radiation during summer, a steep thermocline can form in the upper 20 m, and the intense evaporation cause the surface layer to be more saline. This situation can be reverted with strong monsoonal winds; during the end of the humid season, northerly colder winds promote vertical mixing of the water column. In particular, the SLC can show a vertical and horizontal variability of the water column caused by a combination of events that generate a very dynamic habitat. Among them, upwelling of colder waters from the Gulf of California can cause localized phytoplankton blooms ([31] and references therein), and strong tidal currents [32] can transport bottom sediments and enhance turbidity of the bottom layers of the water column (author FF, personal observation); finally, during the monsoon season, sediment runoff increases due to intense episodic precipitations, decreasing water quality, while atmospheric conditions become more complex (see Appendix A).

### 2.2. Radiative Transfer Equation

4SM relies on the simplified radiative transfer equation (RTE) for optically shallow water (Equation (1)). The RTE has been discussed in detail by Philpot [33] and Maritorena et al. [34]. In this algorithm, the irradiance reflectance of shallow waters below the surface is equal to the deep water reflectance, plus the bottom substrate contrast attenuated by the depth effect. The simplified RTE [34] at the base of the atmosphere (BOA) is rewritten for nadir radiances [23]:

(1) $$\mathrm{BOA}:\;L=\mathrm{Lw}+\frac{\left(\mathrm{LB}-\mathrm{Lw}\right)}{e^{\left(2KZ\right)}}$$

it is inverted as:

(2) $$\mathrm{BOA}:\;\mathrm{LB}=\mathrm{Lw}+\left(L-\mathrm{Lw}\right)e^{\left(2KZ\right)}$$

or at the sensor (Top of Atmosphere, TOA):

(3) $$\mathrm{TOA}:\;\mathrm{LsB}=\mathrm{Lsw}+\left(\mathrm{Ls}-\mathrm{Lsw}\right)e^{\left(2KZ\right)}$$

where L is the spectral BOA radiance where the bottom substrate is at depth Z; Lw is the spectral BOA radiance of the sea where the bottom is optically deep, also defined as backscatter or water volume reflectance; LB is the spectral BOA radiance of the bottom substrate at null depth. By adding the atmospheric path radiance (La) to all BOA radiance terms, one gets the spectral TOA radiances at the sensor LsB, Ls and Lsw in Equation (3). 2K (or K for brevity) is an operational two-way diffuse attenuation coefficient, in units of m^−1^, the sum of the down-welling and up-welling terms together (2K = Kdown + Kup) and Z is the actual depth of the seabed. The optical depth 2KZ, is dimensionless. Wavelength dependency subscripts of all L and K terms are omitted for brevity. This will finally allow for the linearization of the bottom contrast:

(4) X=ln(Ls−Lsw)

4SM is a “ratio method”. For a pair of bands i and j, with K_i_ < K_j_ and suitable bottom contrast in band j, water column correction is achieved by increasing Z in Equation (2) until the ratio LB_i_/LB_j_ matches the slope of the soil line (see the optical calibration section below), where LB_i_ is the average of all bands with K_i_ < K_j_ and suitable bottom contrast. This process operates ratios among relative numbers, and therefore does not require radiance terms to be converted into units of calibrated reflectance. All radiance terms in the above equations represent the signal as measured by the sensor, and the terms digital numbers (DN), radiance and reflectance are used interchangeably and remain dimensionless in this article. This is the privilege of a “ratio method”: the 4SM approach does not require formal atmospheric correction.

### 2.3. Image Resources Preparation

It was soon realized that the hydrological conditions of the La Paz area are complex and highly variable. Therefore, a time-series of Landsat-8 images have been downloaded from https://earthexplorer.usgs.gov/; all scenes were searched with a less than 10% cloud cover condition. Preference was given to images from different months and seasons, avoiding duplicates from the same month, since such a time-detailed study was not the aim of the work. Nine images were selected to be processed and considered reasonably safe to be modeled (Table 1). We processed them using the 4SM method, then using the ENVI/SPEAR tool method.

As regards ENVI/SPEARS, the image preparation and preprocessing started with the creation of vector polygons to be used as masks for clouds and boats. Then, if necessary, the images were de-glinted following Hedley et al. [35]. The de-glinting process assumes a null penetration in water of the NIR-SWIR bands of the spectral images. Areas that are affected by glint are selected over deep water and then are used to calculate linear models of all bands against the NIR or SWIR band. The amount of glint is evidenced and then subtracted using the linear models.

The images are then pansharpened using a modified version of the OrfeoToolbox algorithm in R-project in the Raster Package [36]. In this process, the generation of a high resolution image (PXS) is achieved through the fusion of the panchromatic (PAN) with the multispectral (XS) data. First, the two images need to be orthorectified by oversampling of the XS image, then a low pass filter is applied to the PAN band to equalize (in the Fourier domain) the spectral content with XS data. Once the spectral content is similar, XS are normalized with the low-pass filtered (PAN) and results are multiplied with the original panchromatic band. The whole process can be represented as:

(5) $$\mathrm{PXS}\left(i,j\right)=\left(\frac{\mathrm{XS}\left(i,j\right)}{\mathrm{Filtered}\left(\mathrm{PAN}\right)}\right)\mathrm{PAN}$$

As regards 4SM, the image preparation only consists in importing the raw data as digital numbers in a working database, and creating a few shapefiles for sampling glint, optically deep water, vegetation, and bright sandy beach. Deglinting and pansharpening in 4SM uses the same protocols as above; however, they are applied on the fly rather than as image pre-processing.

### 2.4. Optical Calibration of the Simplified RTE in 4SM

As presented by Morel and Favoretto [23] and at www.watercolumncorrection.com, it is possible to retrieve from the image all the information needed for the calibration of the simplified RTE. Solving for Z and LB in Equation (2) requires four spectral parameters which are estimated from the image itself: (1) the deep water reflectance Lsw; (2) the water column reflectance Lw; (3) the soil line; and (4) the brightest pixels line.

Estimating the TOA deep water reflectance Lsw requires the presence of an area in the image which is free of any glint (or de-glinted), and is optically deep in all wavebands. To estimate the soil line [37], the non-vegetated areas on the land part of the image are analyzed in order to provide the operational values of the ratios LB_i_/LB_j_ of the bottom substrate at null depth for all possible pairs of wavebands i and j. These ratios are then assumed to apply to spectrally non-differentiated shallow water pixels.

Estimating the water volume reflectance Lw is done by visual inspection of the soil line in the optical calibration diagram, and using some commonsense knowledge. Therefore, the atmospheric path radiance La may be estimated as the difference La = Lsw − Lw. Please note that, by subtracting La from all Ls terms in Equation (3), a first order atmospheric correction of the imagery is achieved.

**Figure 2:**
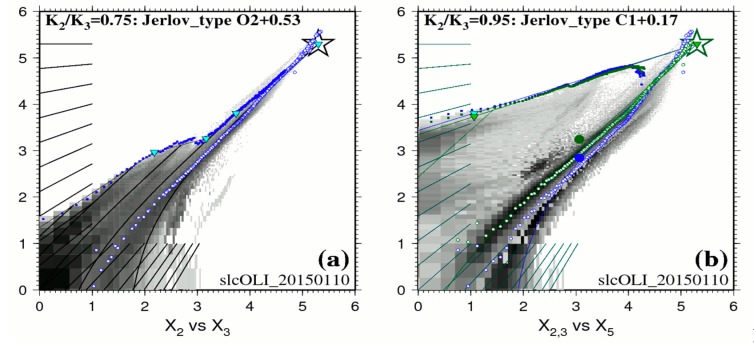
Example of calibration diagram linearized data in logarithmic values for the white ROI of Figure 1. (**a**) is the calibration diagram of linearized Blue (X_2_) and Green (X_3_) and reports estimated K_2_/K_3_ values with Jerlov water types. (**b**) is the calibration diagram for Blue (X_2_), Green (X_3_) and Red (X_5_) bands respectively. The backdrop in shades of gray represents the bi-dimensional histogram for all image pixels (white ROI in Figure 1). Blue dots are the actual pixels used for BPL calibration, each referenced to its row/line position in the image. Small white circles represent the scatter of averaged bare land pixels, as a proxy for the Soil Line.

BPL is a radiometric model of the brightest shallow bottom over the whole depth range in the image. It assumes that the shallow water part of the image contains at least some stretches of the brightest bottom substrate that exists at the scene at various depths; it represents the exponential decay of the radiances as the bottom depth increases. The BPL is used to estimate the ratio K_blue_/K_green_ of the diffuse attenuation coefficients in the blue and green bands. Then this ratio is used to interpolate spectral K for all visible bands, using Table XXVII of Jerlov [12]; this dataset reports the diffuse attenuation coefficients for downwelling irradiance in oceanic and coastal waters worldwide. For two examples of straightforward optical calibration diagrams over apparently homogeneous waters (one oceanic water type, and one coastal water type), please refer to diagrams in Appendix B. For most scenes, the calibration diagrams exhibit a complex hydrologic situation, which we tentatively explain as follows: the clear deep oceanic waters are usually covered by a layer of slightly turbid coastal waters; the thickness of this upper layer varies across the scene, from just a very few meters to in excess of 30 m (see Appendix B). This vertical structure is illustrated in Figure 2 for a Landsat 8 scene acquired on 1 January 2015: X2, X3 and X5 are linearized Blue, Green and Red bands respectively Equation (4).

**Figure 3:**
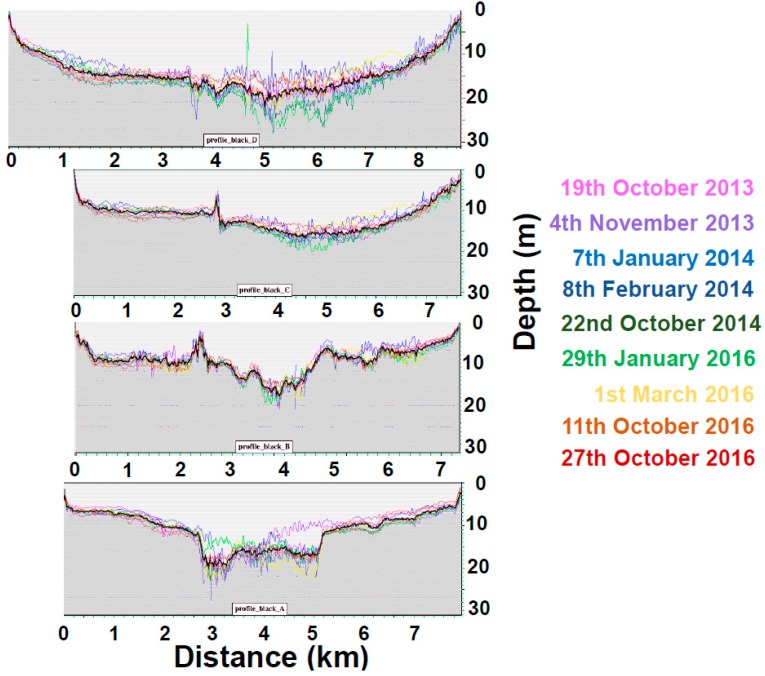
Plot of the depth combination process along four profiles across the SLC. All profiles run from North to South. Thick black profiles are combined depths.

This diagram exhibits two layered water types:

- 0–5 m depth range: on that day, the BPL pixels in Figure 2a clearly display along a straight line over the 0–5 m depth range for the pair Blue/Green. The ratio K_blue_/K_green_ for this surficial layer is estimated at 0.95; this denotes a water type C1 + 0.17 of Jerlov. The BPL pixels in Figure 2b display along two straight lines for the pairs Blue/Red and Green/Red; these two straight lines have virtually the same slope. Diffuse attenuation coefficients in units of m^−1^ are estimated at K_blue_ = 0.272, K_green_ = 0.285, and K_red_ = 0.774. Please note that 0.272/0.285 = 0.95;
- 5–10 m depth range: Figure 2a seems to exhibit a progressive change in water quality over the 5–10 m depth range;
- 10–25 m depth range: on that day, the BPL pixels in Figure 2a clearly display along a straight line over the 10–25 m depth range. The ratio K_blue_/K_green_ for this deeper layer is estimated at 0.75; this denotes a water type OII + 0.53 of Jerlov. Diffuse attenuation coefficients in units of m^−1^ are estimated at K_blue_ = 0.173, K_green_ = 0.232.
- In case of locally increased attenuation coefficient K (phytoplankton), retrieved depth would be under-estimated accordingly, unless a stratified waters model is specified as shown in Figure 2 in the 0–5 m depth range;
- In case of locally increased sediment turbidity (sediment resuspension), water leaving reflectance would be increased accordingly, therefore retrieved depth would be under-estimated, like shown between 4 and 5 km on Profile A in Figure 3.

Finally, through a series of interactive calibration steps, the practitioner now must progressively fine tune manually all these variables in the command line script, until satisfied that a sound consistency has been achieved: the calibration diagrams allow to take control of the calibration process. This completes the set of parameters that are necessary for operating the inverted RTE (Equation (2)) without the need or use of any field data.

### 2.5. Depth Retrieval in 4SM

4SM is a ratio method, meaning that a band ratio is used to derive both Z and a spectral bottom signature. Let LB_j_ be the water column corrected radiance in band j with suitable bottom contrast Ls-Lsw; Let LB_i_ be the average water column corrected radiance of all available wavebands with K_i_ < K_j_ and suitable bottom contrast. The inversion of the model is achieved by increasing the Z term in Equation (2) until the ratio LB_i_/LB_j_ matches that of the soil line. As detailed by Morel and Favoretto [23], for a Landsat-8 or WorldView-2 image, several solutions are available: the NIR solution, the Red solution, the Green solution, and the PAN solution. In practice, the PAN solution is preferred, as it carries several distinct advantages. The outputs are: (i) a raster DTM of the shallow water area in units of meter, and (ii) several rasters of water column corrected wavebands in units of relative radiance, ready for bottom typing (a “low-tide” view of the scene). Some seatruth data can be used for fine tuning the estimation of Z and for a tide correction.

### 2.6. Combining Depths in 4SM

Single scenes are inevitably affected by alien features that impact the result (e.g., boats and their wakes, small clouds and their shadows, algal blooms, local variations of water optical properties, etc.). Therefore, a combined depth image can be produced by averaging all the retrieved depths that satisfy some quality criteria: for each shallow pixel, first an average depth is calculated; then depths that are outside the standard deviation range are excluded and a new average is calculated. This eliminates discrete artifacts without any smoothing. One can see for example in Figure 3 that, on 4 November 2013 (purple profile), a major depth under-estimation affects the southern half of the deep SLC between 4 km and 5 km on Profile A; an increase in turbidity possibly caused this artifact. These profiles also show that the depth combination reduces much of the noise without any smoothing.

### 2.7. Groundtruthed DTM

**Figure 4:**
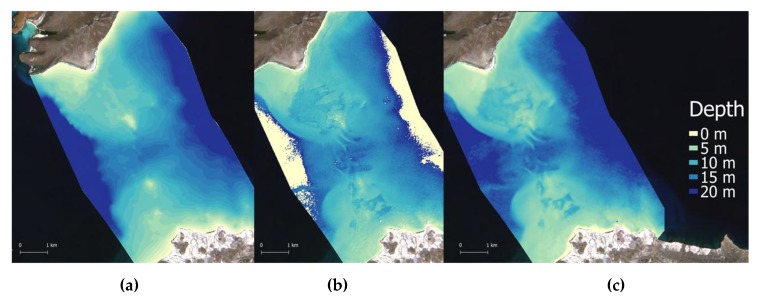
(**a**) is the interpolated DTM of groundtruthed depth points; (**b**) is 4SM satellite retrieved depth layer from 19 October 2013; (**c**) is satellite retrieved depth layer processed with ENVI-SPEAR tool on the same scene.

The collection of ground truth depth soundings in the SLC was completed in two days by the use of a boat equipped with an Elite5x HDI fishfinder (Lowrance, Tulsa, Oklahoma,). The Transducer (TM260 1kW with 50 kHz wide 19o beam/200 kHz narrow 6o beam, Airmar, Milford, NH, USA), was set to read from the waterline. The offset between the antenna and the transducer position was automatically calibrated by the instrument, and the instrument flagged data output with an internal gyroscope in case of excessive wave action. However, during the whole groundtruthing process, sea conditions were excellent. Data have been collected at a constant speed of 10 knots on parallel transects. Up to eight depth readings were averaged for each UTM navigation point, then tide height was subtracted using open source tide data (http://predmar.cicese.mx/calendarios/). Finally, depth data were converted in a shapefile (*n* = 5004, red points in Figure 1), and a Digital Terrain Model (DTM) was created by spline interpolation of groundtruthed depths (Figure 4a).

### 2.8. Depth Retrieval in ENVI-SPEAR Tool 

**Figure 5:**
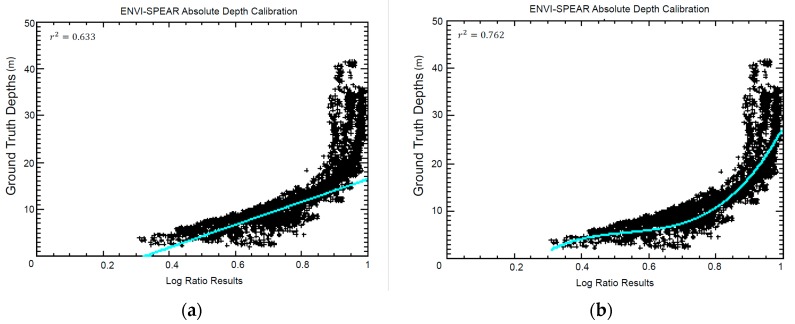
Absolute depth calibration plot calculated with ENVI-SPEAR tool with (**a**) linear model calibration and (**b**) polynomial curve model calibration.

Eight scenes were processed with the ENVI-SPEAR tool over the same ROI as 4SM (Figure 1). This tool operates the Log Ratio Transform algorithm developed by Stumpf et al., [22]. It is integrated in the ENVI software (Exelis Visual Information Solutions, Boulder, CO, USA, v.5.3) as a Spectral Processing Exploitation and Analysis Resource (SPEAR) tool, from now ENVI-SPEAR tool. This tool is capable of retrieving relative depths from multispectral images which then are calibrated into meters using an existing depth sounding dataset. To this end, the groundtruthed DTM described above were used. An example of the output is shown in Figure 4c. Alien features were masked. Then an approximate atmospheric correction was applied on the whole image by Dark Pixel Subtraction. Images were pansharpened, then the Log Ratio Transform algorithm was applied. A 3 × 3 median filter was chosen to remove high frequency noise. Then all groundtruthed depth points (*n* = 5004) were used to calibrate relative depths into absolute depths. In the calibration tool (Figure 5), it is possible to select a model that best fits data based on r^2^ results (linear, exponential, polynomial model). To compare different results, a linear fitting (Figure 5a) and the model with the highest r^2^ were chosen for each scene (e.g., Figure 5b).

### 2.9. Groundtruthing Regressions and Comparisons

**Figure 6:**
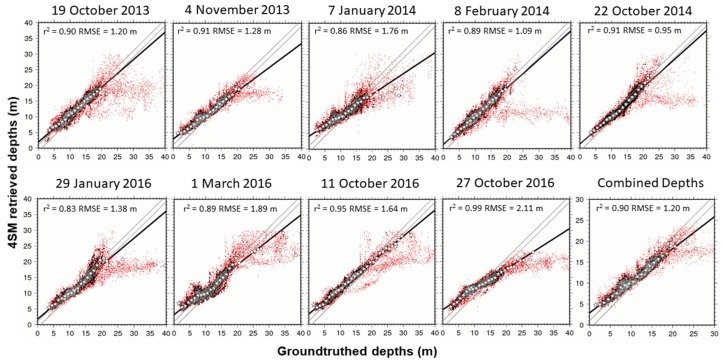
Scatterplots of 4SM retrieved depths vs tide corrected groundtruthed depths for nine scenes, plus combined depth (lower right). Please note the different scale in the combined depths plot.

As regards the accuracy of 4SM depths, pixels with both groundtruthed depths and satellite derived depths are selected and these depth pairs are assigned to bins in decimeters [23]. Bins with few data pairs are excluded as outliers and plotted as red points in the scatterplots of Figure 6. These outliers are generated by various alien features and must be excluded for a fair regression calculation. Then all the remaining bins are averaged into bins in meters.

**Figure 7:**
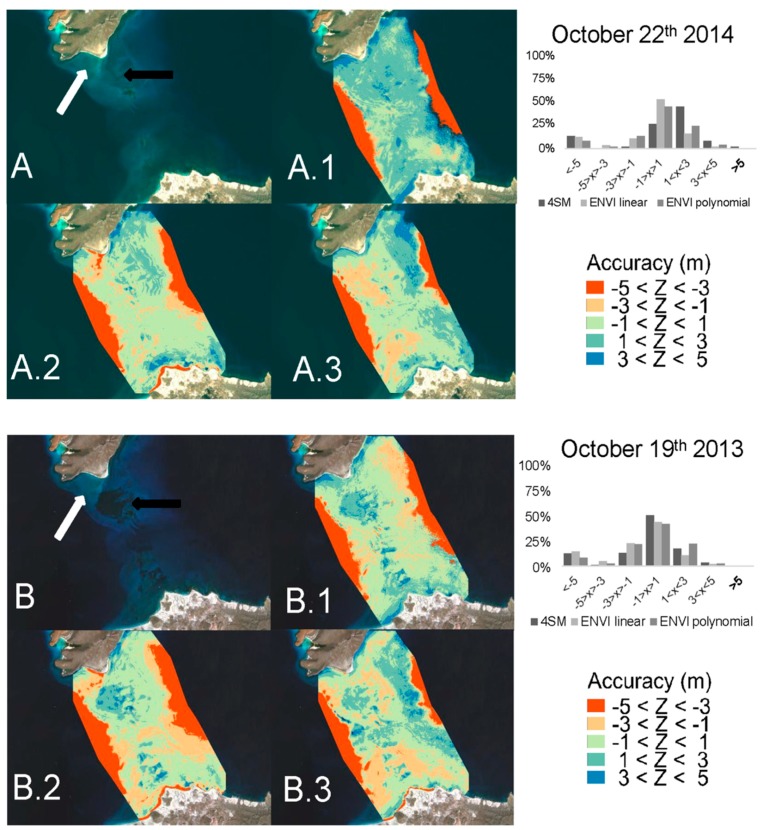
Results of the accuracy assessment on retrieved depth for 4SM and ENVI/SPEAR tool methods. The accuracy index is classified as follow: blue tones for two depth overestimation classes, red tones for two depth underestimation classes, and turquoise tone where the residual is less than 1 meter. Barplots show the percentage of pixels that belong to each of these classes. (**A**) is a Landsat RGB color composite view of 22 October 2014, white arrow indicates an example of bright shallow bottom, while black arrow represents darker shallow bottoms. (**A.1**) is 4SM accuracy index; (**A.2**) is the accuracy index calculated on ENVI-SPEAR with line model calibration; (**A.3**) is the accuracy index calculated on ENVI-SPEAR with polynomial model calibration. Topright barplot reports % of pixels belongings to each index classes: <−5 m difference class is due to the detection limit constraint of remote sensing in coastal water, since it is represented by pixels in deeper waters. (**B**) is a Landsat RGB color composite view of 19 October 2013, (**B.1**) is 4SM accuracy index; (**B.2**) is the accuracy index calculated on ENVI-SPEAR with line model calibration; (**B.3**) is the accuracy index calculated on ENVI-SPEAR with polynomial model calibration. Topright barplot reports % of pixels belongings to each index classes calculated on the scenes.

In Figure 6, all one-meter bins which are represented by a star symbol account for a total of 99% of all accepted depth pairs, while the remaining one-meter bins are represented by a white circle. Inside the two parallel gray lines in the scatterplot, satellite retrieved depths within ± 1 m of groundtruthed depths are included. A correlation coefficient (r^2^) and a Root Mean Squared Error (RMSE) were calculated for each linear model: see Figure 6 and Table 1. Tide heights were adjusted manually and added to seatruth depths in order to minimize the RMSE result. Two scenes stand out with unrealistic tide heights (1.6 m and 1.7 m): this is possibly the result of a bad estimation of the soil line for these two scenes. In order to compare both 4SM and ENVI-SPEAR tool results versus the interpolated DTM of groundtruthed depth points, an accuracy index was estimated by calculating Depth_residual = (Depth_retrieved−Depth_measured). So a positive depth residual signals an overestimated depth, and vice versa. Depth residuals were classified in 5 classes of accuracy, and the percentage of pixels belonging to each class was calculated (Figure 7).

## 3. Results

**Figure 8:**
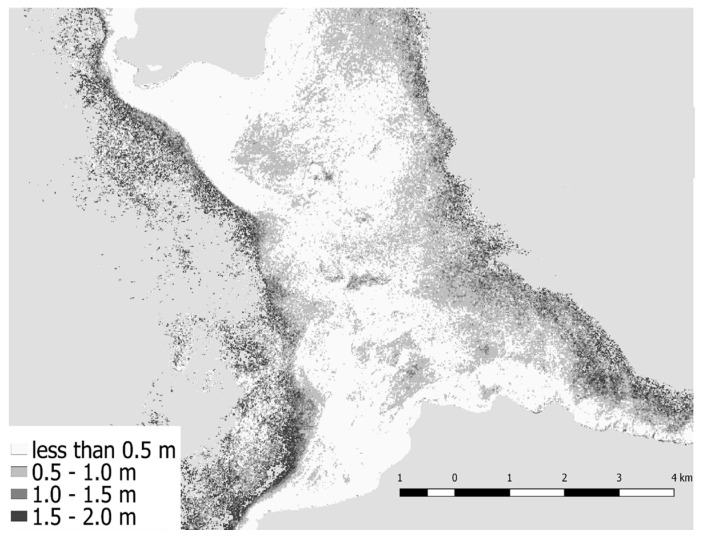
Absolute difference between 4SM retrieved depths and interpolated DTM of groundtruthed depths.

This section first presents the results of the seatruth regressions (Figure 5 and Figure 6), then the results of the accuracy assessment for retrieved depth (Figure 7), and finally the depth residuals (Figure 8).

### 3.1. Seatruth Regressions

As regards 4SM results, linear relationships between retrieved depths and groundtruthed depths are shown for each scene in Figure 6. The regression lines do not account for all depth pairs; red outliers are excluded from the statistics. Linear regression model explained in excess of 80% of the depth variations. The 27 October 2016 scene reports the highest r^2^ (0.99) although it also reports the highest RMSE (2.11 m). The 22 October 2014 scene scored the lowest RMSE. From Figure 6 it is also possible to see how combining depths improved linear fitting results and reduced the noise.

For ENVI-SPEAR tool results, please refer to Figure 5. In Figure 5a a linear model fitting is applied to the scatterplot, while in Figure 5b the best model that showed the higher r^2^ is plotted. In particular, for eight ENVI-SPEAR scenes regression plots, the highest correlation coefficient r^2^ was always achieved with a polynomial model.

### 3.2. Accuracy Assessment

**Figure A1:**
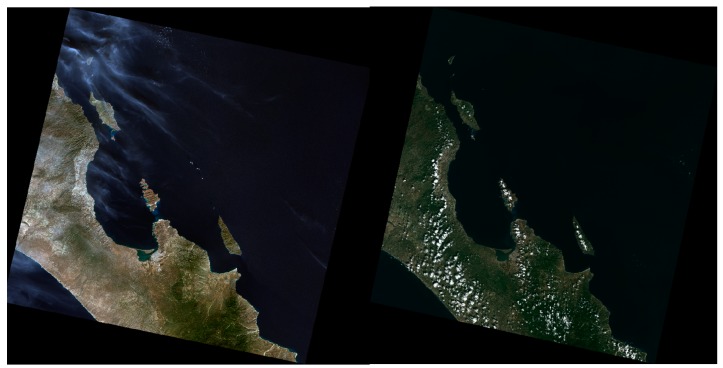
Example of complex atmosphere conditions.

**Figure A2:**
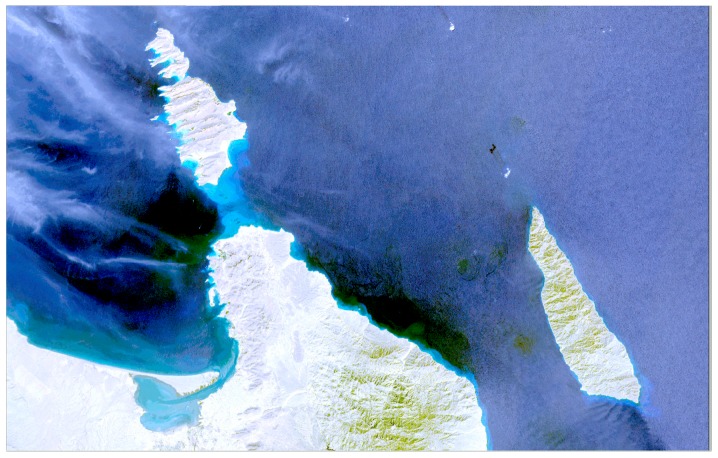
True color composite of the 27 October 2016 image. This scene exhibits a complex atmosphere and distinct blooms of discolored waters.

**Figure A3:**
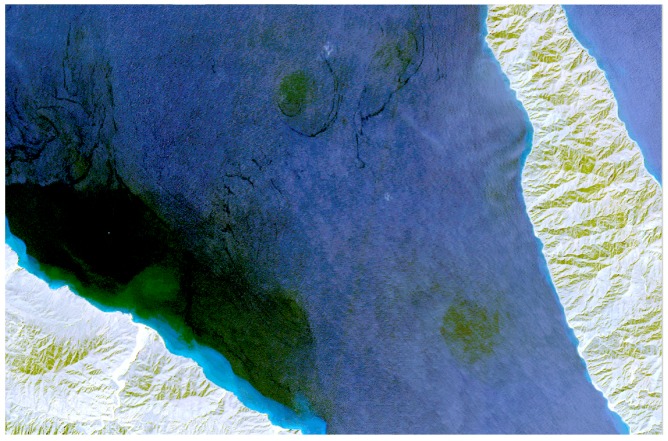
True color composite of the 27 October 2016 image. This scene exhibits distinct blooms of discolored waters (algal blooms).

Accuracy assessments are presented in Figure 7. The accuracy index allowed a visual estimation of overall accuracy of the retrieving methods applied for each scene. In terms of % of accuracy within ±1 m of groundtruthed depths, 4SM performed better in 4 out of 9 scenes; in the 3 remaining scenes, it scored similar results with ENVI-SPEAR with the exception of one scene: 22 October 2014 (Figure 7A.1 and black bars in linked barplot chart) that is reported to be further discussed. In Figure 7B, 19 October 2013 scene is reported because 4SM scored its best results (50% of pixel within ±1 m of groundtruthed depths, Figure 7B.1 and black bars in linked barplot chart). Over shallow bright bottoms, 4SM performed better than ENVI-SPEAR (Figure 7A,B white arrows); in particular, with a linear fitting model, ENVI-SPEAR tends to underestimate depth by more than 3 m over bright bottom areas (Figure 7A.2,B.2). It is also observed, in Figure 5a, that linear fitting of ENVI-SPEAR results exhibits a detection limit: deeper than ~15 m, most retrieved depths are not correlated to real depths anymore; the polynomial model exhibits a better fit because of the curved shape of scatterplots above 0.8 Log Ratio results (Figure 5b). Moreover, in Figure 7A.1–A.3, it is observed, that both 4SM and ENVI-SPEAR tend to overestimate depths over dark bottoms at shallow depths. Finally, except for 22 October 2014 and 27 October 2016 which both exhibit complex atmospheric and hydrologic conditions (Appendix A, Figure A1, Figure A2 and Figure A3), accuracy of the methods is similar. Therefore, in spite of the adverse conditions of the SLC, and without the need of any field data, the 4SM method is comparable to the ENVI-SPEAR method, and equally valid.

### 3.3. Depth Residuals

Depth residuals are defined by the absolute difference between combined 4SM depths and DTM. Depth residuals are represented graphically in Figure 8. Most of the depth residuals were ≤0.5 m, especially over bright shallow and deeper (≈15 m) substrate, where 4SM showed more consistency. Conversely, higher residuals were found in two areas corresponding to darker bottoms (see also Figure 7A,B black arrows). Over time this area showed a change in bottom properties, when in 22 October 2014 a large portion of a low albedo patch on the northern side disappeared and wasn’t recorded anymore. Finally, it is evident how areas below 20 m were the ones that showed higher residuals, where depth retrieval reaches its optical limit in this environment.

## 4. Discussion

### 4.1. San Lorenzo Channel’s Conditions

The results suggest that 4SM can be safely used on single scenes under reasonable conditions of low cloud cover, low glint effects, and homogeneous vertical and horizontal water optical properties. The SLC did not show any relevant variations in depth along the four years analyzed. However, the area was characterized by high seasonal variations in vertical and horizontal stratification of the waters; the same can be said for variations of spectral characteristic of the bottom, that are currently under investigation (Favoretto et al., unpublished data).

**Figure 9:**
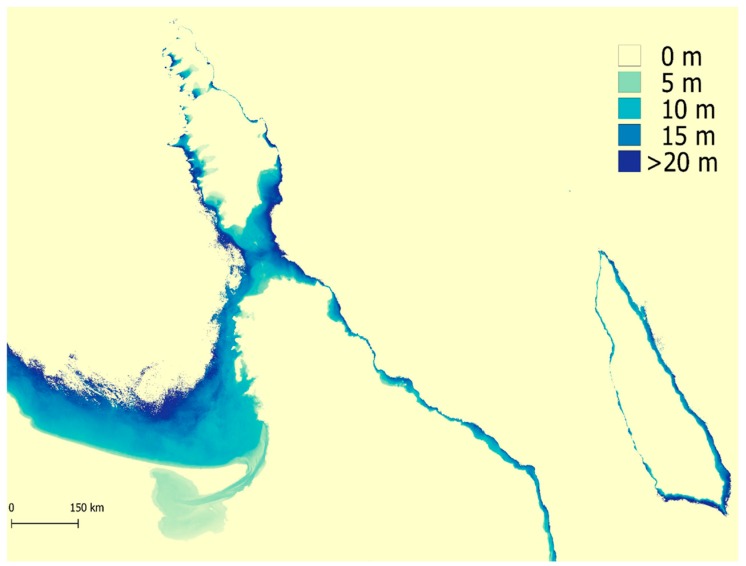
4SM is capable of calculating bathymetry values without field data and this is the results of satellite derived bathymetry for the whole La Paz bay.

The application of 4SM not only allowed the retrieval of the bathymetry of the SLC that showed to be consistent with groundtruthed depths, but it generated a bathymetry model for the whole shallow area in the Bay of La Paz (Figure 9). In particular, the SLC showed a fair amount of layered waters with seasonal and spatial complexity, and 4SM does account to a limited extent for such layered waters while calibrating the simplified RTE. Overall, waters in the channel were vertically stratified, with common algal blooms events (red tides) in the complex hydrodynamic conditions in the Bay of La Paz ([31] and references therein). In the 22 October 2013 scene for example, deep oceanic waters are covered by an upper layer of Coastal waters: in the San Gabriel bay (Figure 1, red square), this upper layer is ~5 m thick, but it is ~9 m thick in the SLC. Such heterogeneity is commonly found through the seasonal cycle for the scenes investigated. All of this considered, it is possible to obtain useful information even from low quality areas in the scenes, accounting for their limitations.

### 4.2. Models Comparison

In Figure 7A,B, RGB color composite present the worst (A) and the best (B) scenes in terms of 4SM accuracy. It is important to note a major change in bottom composition from 2013 that clearly show a dark patch (black arrow Figure 7A) of what probably was macroalgae (Favoretto et al., unpublished data) that disappeared in 2014, and up to now it has not been detected again. This change may have been caused by Hurricane Odile in September 2014, that displaced significant amounts of sediments (Favoretto et al., unpublished data). This event allowed for a comparison between low-albedo bottom processing capabilities of 4SM and ENVI-SPEAR. The bottom albedo independent nature of ENVI-SPEAR (Stumpf’s) algorithm should guarantee in some limits that dark bottoms are shown at the same depth as bright sand, when field data report so. However, the method applied in the SLC shows how depths over dark bottoms are overestimated within 5–10 m depth range (Figure 7B.2,B.3). In particular, the linear model calibration of ENVI-SPEAR algorithm yields higher depth residuals than the polynomial model calibration. (Figure 7A.2,A.3). This limitation that was already pointed out in the introduction of this article about Stumpf’s algorithm. As the results suggests, 4SM perform better over very bright shallow areas, but has the same problems as ENVI-SPEAR over very dark bottoms (Figure 7B.1).

In the 3–10 m range, retrieved depths are over-estimated by 4SM: this means that bottom substrates are actually less green than assumed by the Soil Line in 4SM. In this depth range, dense red coralline algae formations, called rhodolith beds, characterize the area [25,26]. Coralline algae absorb additional wavelength over the green range for their photosynthetic activities, and their common presence in the area could in part explain a lower than expected green reflectance. This hypothesis is currently under investigation (Favoretto et al., unpublished data).

Over the 10–20 m range, most retrieved depths are under-estimated by 4SM: this means that bottom substrates are actually greener than assumed by the Soil Line in 4SM. This depth range is present in the SLC exclusively in its central portion, where tidal currents are supposed to merge in stronger bidirectional deeper currents. In this portion of the channel, where strong currents shape the sandy bottom: cyanobacterial biofilms can cover large portions of the seabed (Favoretto et al., in prep). These organisms, stretching for meters, can have a significant production and affect the green reflectivity of the substrate in the green band (see Appendix D).

### 4.3. The 4SM Method

In 4SM the detection of the shallow bottom in more than two wavebands allows for a better estimation of Z, and therefore yields a richer spectral bottom reflectance associated to the pixel. To this extent, and from many tests made in the development of 4SM (see www.watercolumncorrection.com, and [23]) it can be said that 4SM is not site-specific, and is capable of delivering both a bathymetric map and a water column corrected image in units of image’s DN over many different water types, in different location of the world, when the high cost of collecting similar data in the field is simply not affordable. While an empirical method (e.g., [21]) explores the statistical relationship between image pixel values and field measured depths, 4SM operates a radiance inversion approach which does not use any field measured water depths for calibration. Using 4SM there is no need for a formal atmospheric correction since the spectral path radiance and the water volume reflectance are estimated through the calibration process. All of these advantages reduce artifacts and enhance operability, simplifying image processing and interpretation.

For each shallow pixel, the 4SM results are an estimate of water depth, and an acceptable fit of the water column corrected spectral bottom signature with the Soil Line, although contrasted bottom signatures entail potentially severe bias on retrieved depth. Another advantage of the 4SM method is the pansharpening adaptation that generates 15 m resolution images to be modelled. Using this modified pansharpening algorithm, pixels which are brighter than their surrounding in the PAN band get a boost in the multispectral band and vice versa. Other empirical methods use only multispectral bands, and this is applicable as long as there is enough contrast in colors (e.g., K_blue_ < K_green_ < K_red_) like in clear oceanic waters. However, in coastal waters, this is not the case and there is hardly any color separation between K_blue_ and K_green_. This actually is a fundamental limitation which precludes a reliable estimation of the model parameters for Coastal water types in methods like Lyzenga’s and Stumpf’s. The PAN solution offers a valuable alternative, as it will always generate enough color contrast, even with Coastal type waters. In the end, 4SM uses the image metadata to convert spectral water column corrected bottom signatures into calibrated reflectance (0−1), ready for bottom typing and time series studies. The method produces a low tide view of the shallow areas along with a DTM; this is a unique feature of 4SM, apart from semi-analytical methods [10].

Moreover, the application of combined depths improved model fitting results; however, it did not improve accuracy compared to other scenes. Strong depth residuals (>2 m) can hardly be interpreted in this case as caused by natural or anthropic events; they are likely noise in depth retrievals due to local perturbations like atmospheric or hydrological effects, but also phytoplankton blooms, turbid waters, wind or even boats wakes. Therefore, any pixel that shows this kind of variation should not be considered in the overall depth model. Combining depths is an important tool in reducing these types of noise (that are inevitable for the practitioner). The fact that most of the pixels depth residuals less than 0.5 m (Figure 8) in the 0 to 20 m depth range is supports 4SM as a robust depth retrieval method that can cope, to a limited extent, with a layered structure of the water column.

## 5. Conclusions

It is possible to conclude that:

- 4SM is independent on field data to achieve the optical calibration;
- it offers a simpler operational flowchart compared to previous methods (e.g., [16,19,22]);
- its accuracy is equally valid, and in some case better, compared to the accepted and widely used Stumpf’s algorithm;
- it provides a priori important insight on the optical properties of the water column (spectral K, i.e., water quality), and also on the hydrological conditions;
- it uses all bands with significant bottom detection and delivers computed depths, and water column corrected spectral bottom reflectance;
- it provides all valuable information that allows to explore and monitor large coastal areas in a more efficient and cheaper way in terms of resources and time.

These results highlight the progress accomplished by 4SM as a band ratio method for satellite derived bathymetry, especially if considering its the independence from any field calibration, and show how the 4SM algorithm can be a relevant alternative to traditional ratio-methods.

## Figures and Tables

**Table 1:** Landsat 8OLI scenes codes processed in this study, with seatruth regression for 4SM retrieved depths.

Scene File Code	Date	Tide (m)	r^2^
LC80340432013292LGN00	19 October 2013	0.0	0.90
LC80340432013308LGN00	4 November 2013	0.6	0.91
LC80340432014007LGN00	7 January 2014	0.0	0.86
LC80340432014039LGN00	8 February 2014	0.6	0.89
LC80340432014295LGN00	22 October 2014	1.6	0.91
LC80340432016029LGN00	29 January 2016	1.7	0.83
LC80340432016061LGN00	1 March 2016	−0.3	0.89
LC80340432016285LGN00	11 October 2016	−0.2	0.95
LC80340432016301LGN00	27 October 2016	0.0	0.99

**Figure A4:**
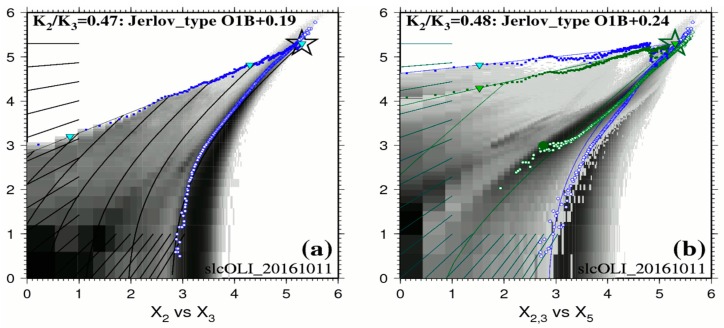
Plot of linearized calibration data (Equation (4)) for the whole of the 11 October 2016 scene. Bands 2, 3 and 5 are Blue, Green and Red, respectively. (**a**) is the calibration diagram of linearized Blue (X_2_) and Green (X_3_) and reports estimated K_2_/K_3_ values with Jerlov water types. (**b**) is the calibration diagram for Blue (X_2_), Green (X_3_) and Red (X_5_) bands respectively. The backdrop in shades of gray represents the bi-dimensional histogram for all image pixels (white ROI in Figure 1). Blue dots are the actual pixels used for BPL calibration, each referenced to its row/line position in the image. Small white circles represent the scatter of averaged bare land pixels, as a proxy for the Soil Line.

**Figure A5:**
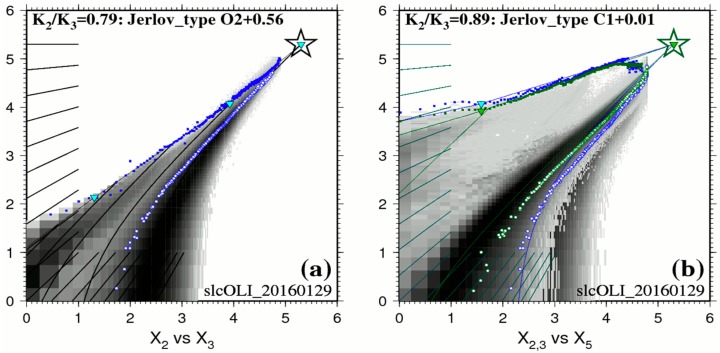
Plot of linearized calibration data (Equation (4)) for the whole of the 29 January 2016 scene. Bands 2, 3 and 5 are Blue, Green and Red, respectively. (**a**) is the calibration diagram of linearized Blue (X_2_) and Green (X_3_) and reports estimated K_2_/K_3_ values with Jerlov water types. (**b**) is the calibration diagram for Blue (X_2_), Green (X_3_) and Red (X_5_) bands respectively. The backdrop in shades of gray represents the bi-dimensional histogram for all image pixels (white ROI in Figure 1). Blue dots are the actual pixels used for BPL calibration, each referenced to its row/line position in the image. Small white circles represent the scatter of averaged bare land pixels, as a proxy for the Soil Line.

**Figure A6:**
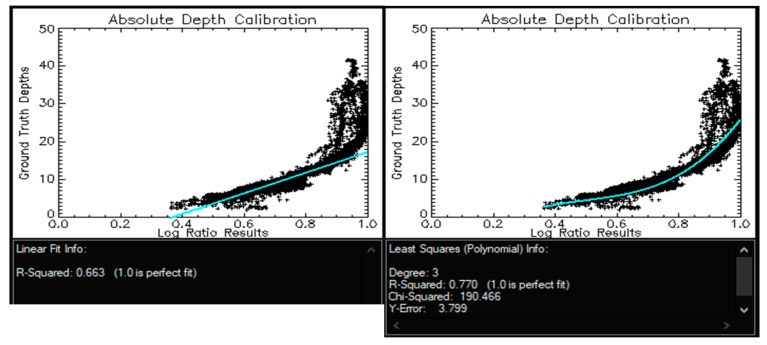
From 19 October 2013.

**Figure A7:**
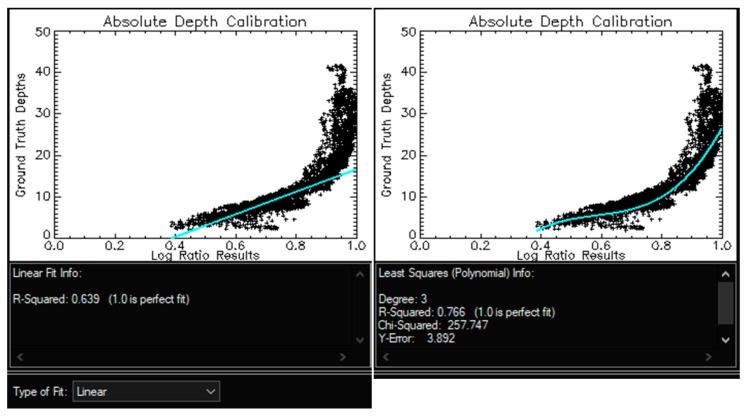
From 4 November 2013.

**Figure A8:**
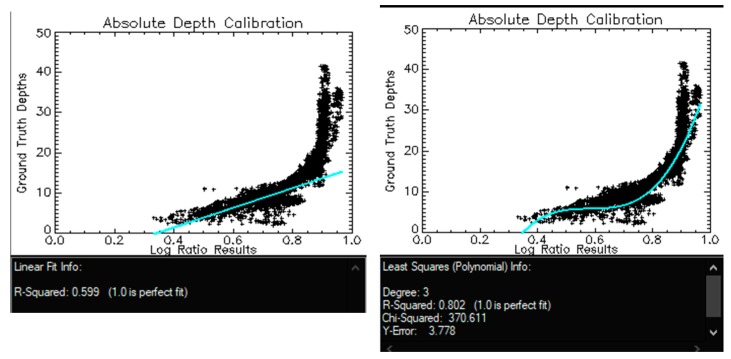
From 7 January 2014.

**Figure A9:**
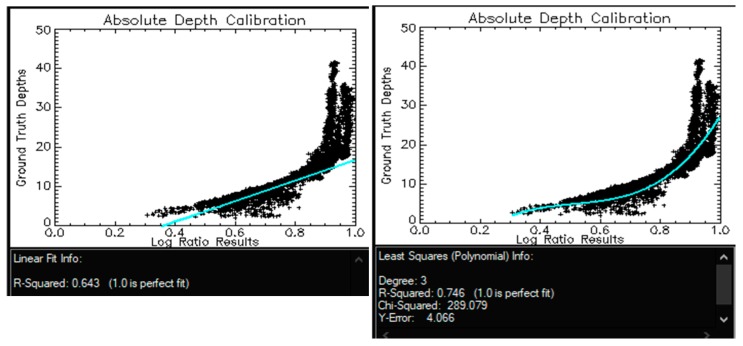
From 8 March 2014.

**Figure A10:**
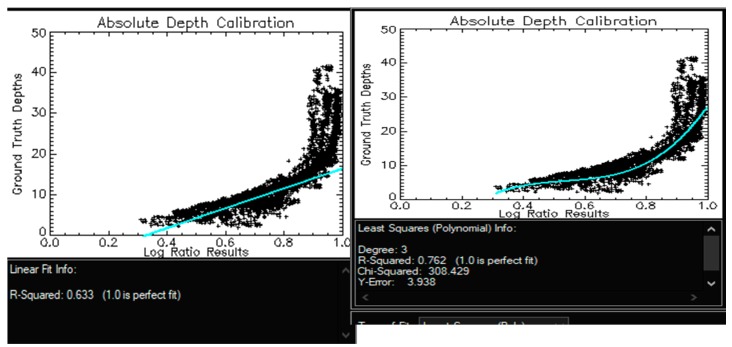
From 29 January 2016.

**Figure A11:**
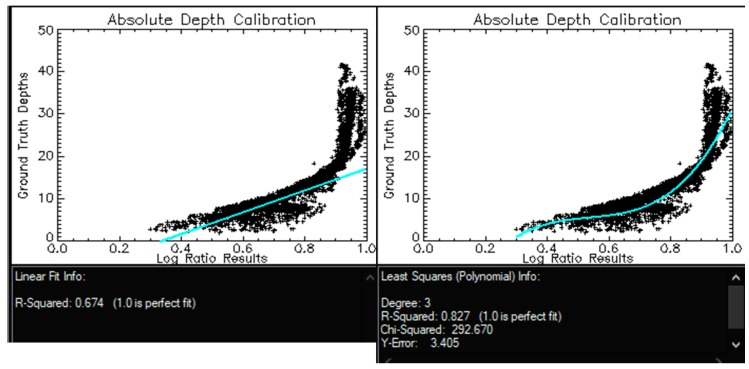
From 1 March 2016.

**Figure A12:**
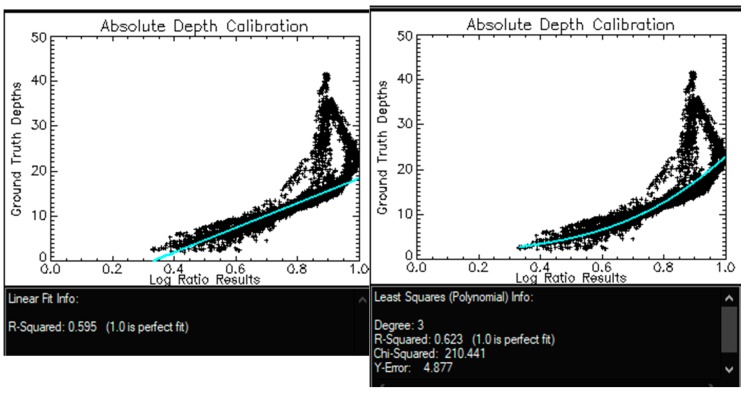
From 11 October 2016.

**Figure A13:**
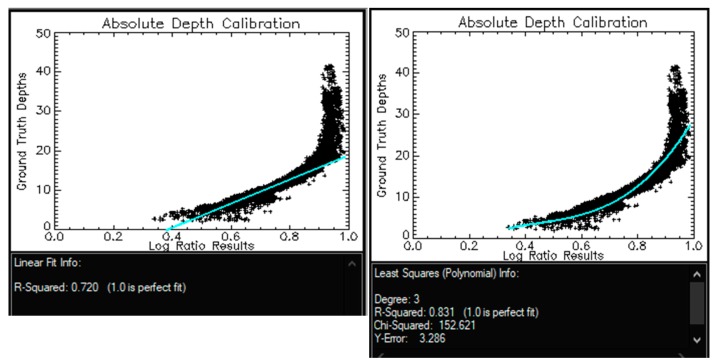
From 27 October 2016.

**Figure A14:**
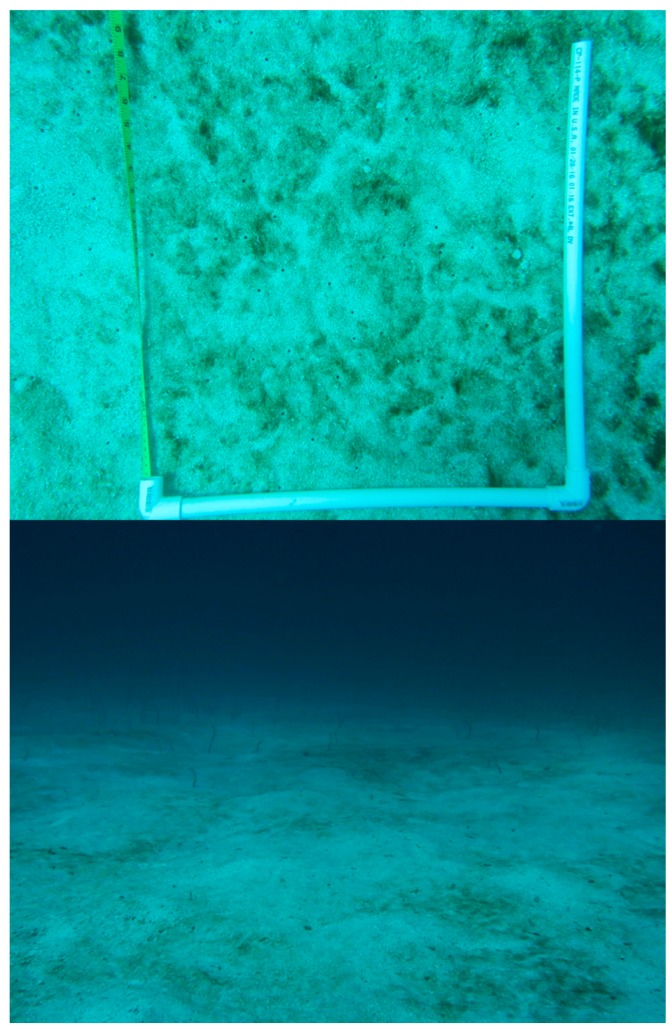
Underwater picture of SLC relevance of cyanobacteria mats over sandy bottoms (Favoretto et al., unpublished data).

## Appendix B

Appendix B: two examples of clear-cut calibration diagrams. In Figure A4 and Figure A5, X2, X3 and X5 denote the linearized Blue, Green and Red bands respectively (Equation (4)). The backdrop in shades of gray represents the bi-dimensional histogram for all image pixels. Blue dots are the actual pixels used for calibration of the BPL, each referenced to its row/line position in the image. Small white circles represent the scatter of averaged bare land pixels, as a proxy for the soil line.

The 11 October 2016 scene offers a nice and straightforward optical calibration diagram for very clear waters. In Figure A4a, the BPL pixels (blue dots) display along a straight line over a depth range of ~40 m; the slope of this line is the ratio K_blue_/K_green_ = 0.47. In Figure A4b, the BPL pixels (blue and green dots) display along two straight lines over a depth range of ~5 m, with slopes K_blue_/K_red_ = 0.103 and K_green_/K_red_ = 0.219. Please note that 0.103/0.219 = 0.47. Using this value to interpolate spectral K from Jerlov’s data yields the following values in units of m^−1^: K_1_ = 0.075, K_2_ = 0.083, K_3_ = 0.174 and K_5_ = 0.651. This case is misleading though, as the optical properties of the waters in this area exhibit much variability, both horizontally and vertically.

The 29 January 2016 scene offers a nice and straightforward optical calibration diagram for Coastal waters of Jerlov. In Figure A5a, the BPL pixels (blue dots) display along a straight line over a depth range of ~30 m; the slope of this line is the ratio K_blue_/K_green_ = 0.79. Using this value to interpolate spectral K from Jerlov’s data yields the following values in units of m^−1^: K_1_ = 0.191, K_2_ = 0.186, K_3_ = 0.234 and K_5_ = 0.878. Please note that, if K_blue_ = K_green_, the ratio K_blue_/K_green_ = 1.0 and there is no color separation at all by diffuse attenuation between these two bands: many shallow pixels would display as a function of noise. Therefore, unlike the Pan solution, which achieves good color separation [23], we can expect that the Green solution would yield bad depth results for coastal waters. In Figure A5b, the ratio K_blue_/K_red_ = 0.33 and the ratio K_green_/K_red_ = 0.37. Please note that 0.33/0.37 = 0.89: the upper layer in the 0–5 m depth range might be slightly less clear.

## Appendix C

Eight ENVI-SPEAR calibration plots are displayed. Left is linear fitting, right is the best model fitting according to the coefficient of determination r^2^.
